# Principal Accompaniment in Australian Faith-Based Schools: A Salutogenesis Approach

**DOI:** 10.1007/s10943-023-01980-8

**Published:** 2024-02-22

**Authors:** W. Sultmann, D. Hall, J. Lamb

**Affiliations:** 1 La Salle Academy, Australian Catholic University, 1100 Nudgee Road, Banyo, 4014 Brisbane, Australia; 2 La Salle Academy, Australian Catholic University, 2060 Sydney, NSW Australia; 3 La Salle Academy, Australian Catholic University, 1100 Nudgee Road, Banyo, 4014 Brisbane, QLD Australia

**Keywords:** Principal, Well-being, Salutogenesis, Accompaniment, Mind, body, and spirit awareness

## Abstract

A program designed to provide accompaniment reflects a salutogenesis emphasis aimed at sustaining the professional well-being of experienced principals. A mixed methods pilot study focused on participant (*N* = 12) orientation, principles of accompaniment, mission-aligned processes, leadership agency, structured conversations, and nominated outcomes. Data were collected over twelve months at three stages using online survey. Australian Catholic principals reported a positive orientation experience, professional well-being, the comprehensiveness of the program, and the manageability of its implementation. Discussion confirmed design elements contributed to the wholistic nature of accompaniment linking body, mind, and spirit in conversational processes and the transformative effects of these exchanges on professional practice and well-being.

## Introduction

The workforce of school principals in Australia parallels global trends (Organization for Economic Co-operation and Development [OECD], 2020) which demonstrate challenges to recruitment, retention, and wholistic well-being (See et al., 2023). Sources of stress for principals entail the quantity of work, the lack of time to focus on teaching and learning, the mental health issues of students, and the expectations of the employer (Independent Education Union, South Australia, 2022, p. 1). Coupled with the reality of long working hours is the stress activated from within the school community with more than 40% of principals reporting threats of violence or being a victim of physical violence, circumstances that increased over the 10-year lifespan of the survey. The combined effect of professional and community stressors is evidenced in a decreasing number of applicants for principal positions, premature retirements, and a growing collegial awareness of the demanding nature of the profession (Riley, 2016; Watts, 2022).

Catholic school principals in Australia are also impacted by the vocational, professional, social, and cultural influences which challenge personal well-being and influence leadership (Branson et al., 2019). In this context, well-being is conceived as a state of striving for and/or achieving positive bio-psychosocial and spiritual potential. It is a concept that emphasizes life as wholistic and essentially comprehensible, meaningful, and manageable. The importance of health and well-being is summarized in the concept of salutogenesis as a means of understanding and intervening in circumstances of challenge. Derived from the Latin words “salus” meaning health and “genesis” meaning origin, salutogenesis focuses on the creation and maintenance of holistic health in contrast to a pathogenic model focusing upon existing diseases (Simons & Baldwin, 2021). The model of salutogenesis entails proactive and preventative responses to manage stress through intentional awareness and engagement with the physical, social, emotional, spiritual, intellectual, vocational, and environmental dimensions of the principal. Principles which underpin a salutogenic approach focus on interventions which are respectful of the health support continuum, engage the story of the individual, support ill-health prevention, and address stressors as means for enabling personal adaptations (Mittelmark, 2021). Salutogenesis provides for health and well-being by strengthening socio-ecological health resources, promoting a sense of coherence through helping groups and individuals to comprehend and make sense of one’s own experiences, and the ability to manage and respond flexibly to the inevitability of life stressors (Mittelmark, 2021, p. v).

Leadership within Catholic schools is unique in that leadership is situated within the story and experience of the individual and the history and contemporary context of the Catholic school. It is not only mindful and responsive to professional challenges and social circumstances but is shaped and guided in these circumstances by beliefs and values centered in the Catholic tradition (Sultmann, 2018) and the integration of faith, with life and culture (Congregation for Catholic Education, [CCE] 1977, para 37). As with principals generally, Catholic school principals also experience stress through “increased administrative and managerial workloads, requirements to communicate and engage with the community, and pressure to meet or exceed standards have made the principalship more time-consuming and complex” (Watts, 2022, p. 1). The situation is such that support to principals takes on a new level of priority with well-being as core and interventions that are supportive of outcomes which value add to meaningfulness, comprehensibility, and manageability in ways that integrate the social, physical, spiritual, and intellectual dimensions of the role (Mittelmark, 2021).

## Leadership Support Through Accompaniment: Mamre

Recognition of the complexity and challenge of school leadership underpinned the motivation of an Australian Catholic education authority to develop, implement, and evaluate a leadership support initiative based on accompaniment. The pilot project entitled *Mamre: Leadership accompaniment in Catholic schools* was characterized by leadership as informed by the Spirit, vocational in nature, and collegial in practice. As a complement to other system support processes, Mamre was conceived as a mutually supportive dialogical process which recognized school context and culture, the agency of the principal, and the expertise of the companion who accompanies the principal. The initiative was invitational in nature and founded on the intentions of strengthening commitment and well-being for mission in the Catholic school. As a pilot initiative, it was imaged through the Biblical account of the experience of Mamre. Mamre recalls the historical place of Mamre, an ancient religious site located on a hill on the route between Hebron and Jerusalem, a meeting place where engagement unfolded and dialog flourished. According to the Biblical account, Abraham pitched his tent there, under an oak tree (Gen 13:18), and is said to have encountered three angels. Mamre is characterized as a place of rich and sacred engagement, a place of hospitality and fruitfulness enriched by the Spirit, and a place which offers an image of what accompaniment in the tradition of faith might look like. It preempts the road to Emmaus and is contemporized and applied by Pope Francis in processes of dialog and the richness of discernment (Rixon, 2019). “We need a church capable of walking at people’s side, of doing more than simply listening to them; a church that accompanies them on their journey” (Francis, 2013b, p. 1).

### Theologizing Accompaniment

Christian scriptures identify God as love (1 John 4:8), and it is out of God’s love that humankind has come to an understanding that “God’s very being is truth and love” (Catechism of the Catholic Church, [CCC], 1994 para. 231). Through love, God freely and reciprocally interrelates with all creatures in a manner that respects their dignity and core identity. In this way, all people are related to one another, and the divine presence, incarnation, can be seen in all things (CCC, 1994, para. 41). Action in the Spirit of love is identified in *The Joy of the Gospel* (Francis, EG, 2013a, paras. 261–283) as a missionary impulse. It is living the Gospel in practical form, being responsive to the Spirit and seeking alignment with the person and message of Christ. It is the Spirit that brings grace, God’s love, to all human activity and leads to liberation, transformation, and salvation. In this way, Word and Spirit, Christ, and the action of the Spirit make explicit the imagination of Christ in the life of the Catholic school.

Catholic school leadership, grounded within a view of God with us, is “inductive, earthed, and makes Christ’s love visible in the midst of ordinary human life” (Maher, 2015, p. 19). Accompaniment which supports this leadership is also grounded in faith and expressed in a relationship of service and communion underpinned by love and truth. Accompaniment is a living expression of belief, through relationship, within the reality of the ordinary and the not-so-ordinary experience of the principal and the companion. It is introspection, contemplation, and sharing of wisdom lived out in the Spirit of incarnation as bringing God into the foreground of awareness in ways that illumine experience and give life for and within all. Accompaniment in the Spirit of faith is viewed as an experience “which teaches us to remove our sandals before the sacred ground of the other (cf. Ex 3:5); and in the process to be steady and reassuring, reflecting our closeness and our compassionate gaze which also heals, liberates, and encourages growth in the Christian life” (Francis, 2013a, n. 169).

The process of accompaniment between the principal and the companion opens channels of understanding through gentle and respectful listening. Enhancing professional effectiveness and personal well-being is dependent upon the quality of the relationship, the richness of the exchanges between the participants, and the value of the reflection (Topliss, 2020). The literature from the Congregation for Catholic Education; *Educating to Intercultural Dialogue in Catholic Schools Living in Harmony for a Civilization of Love* (CCE, 2013) and *Educating to Fraternal Humanism* (CCE, 2017) speak of this relationship as centered in dialog, reflecting the person and message of Christ, and expressed in communities where relationships are pervasive and foundational to mission. It is the art of becoming attentive to the Divine in the ordinary and not-so-ordinary through being present within encounters and developing a sense of the work of the Spirit (Sultmann, 2018).

### Accompaniment Characteristics

Leadership accompaniment serves personal, professional, and organizational outcomes (Holmes, 2023). It is characterized by a relationship founded in companioning. Companioning in the tradition of the Jesuit order is a practice that involves providing support and guidance to people in their spiritual journey. Jesuit companioning is grounded in the principles of Ignatian spirituality which emphasizes the importance of discernment, service, and mission (Marek & Walulik, 2022). The Jesuit tradition also reinforces the importance of building relationships and engaging with others in a meaningful way (Yevenes, 2005) in support of one’s personal spirituality. The understanding of spirituality, adapted from Rossiter (2015), is the natural dimension to life that includes thinking and feeling about transcendence; human values; love and care for self and others; and sense of stewardship for the earth and its flora and fauna. In summary, it is “the consistent application of one’s spirit to the circumstances and relationships of life and living” (Sultmann, 2018, p. xii). Notwithstanding these overall emphasizes, companioning informed by the spirit of Mamre reinforces the Spirit in mission, as faith informs life and culture and the process of companioning nurtures the mutual transformation of the principal and the companion.

A key characteristic of companioning is the practice of discernment. Discernment involves the process of making decisions in a way that is guided by the Spirit and is in line with God’s will (Rixon, 2019). This process entails prayer, reflection, and consultation with others, and within the Mamre program, it is a fundamental component of the encounter between leader and companion. Companioning involves helping leaders to discern their own call to service and to work toward the greater glory of God. This involves listening to others, being present to them, and offering support and guidance in working toward the common good (Yevenes, 2005). The practice is one of engaging comprehensively in matters relevant to the leader.

**Fig. 1:**
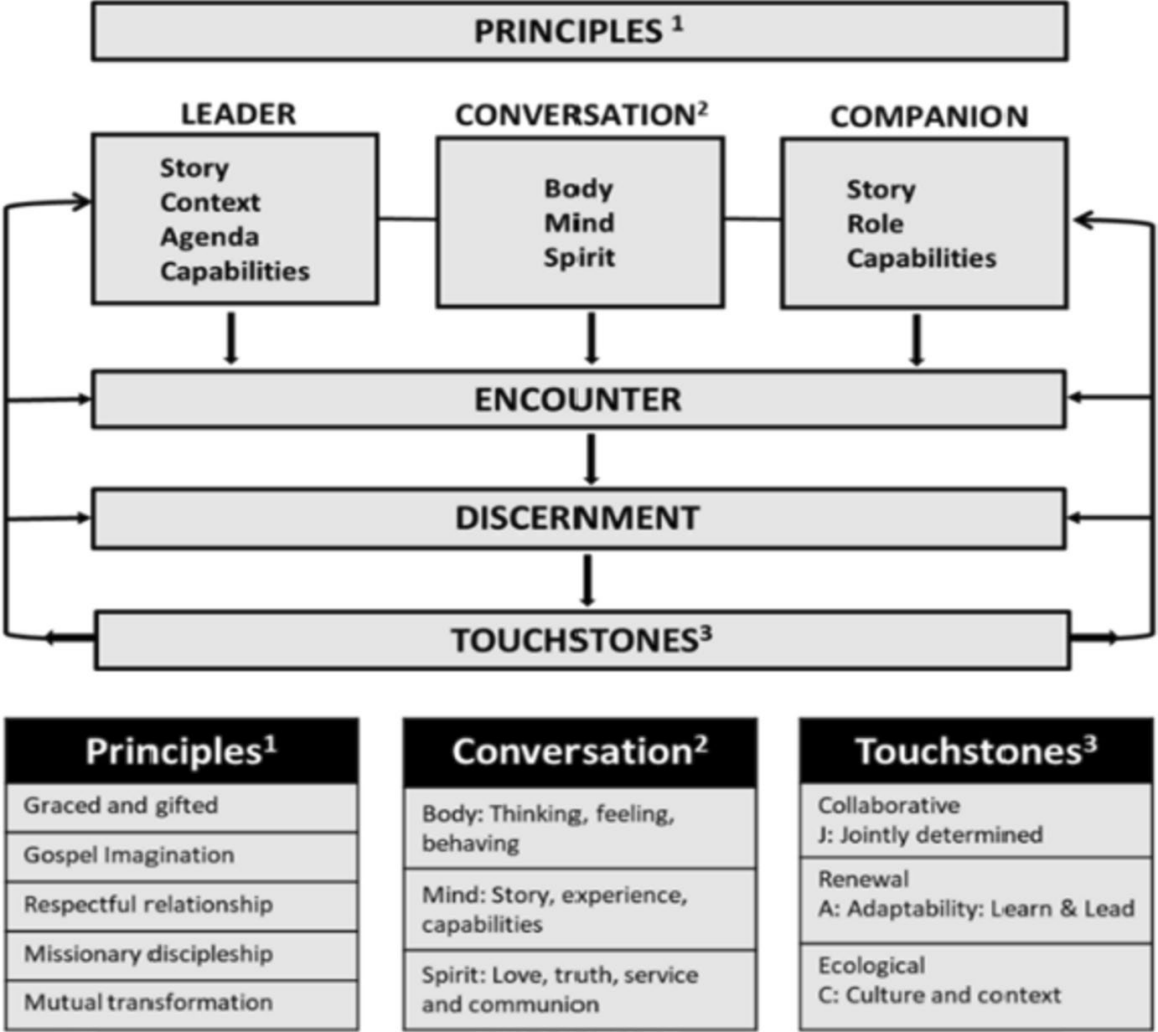
Summary of the accompaniment process

**Fig. 2:**
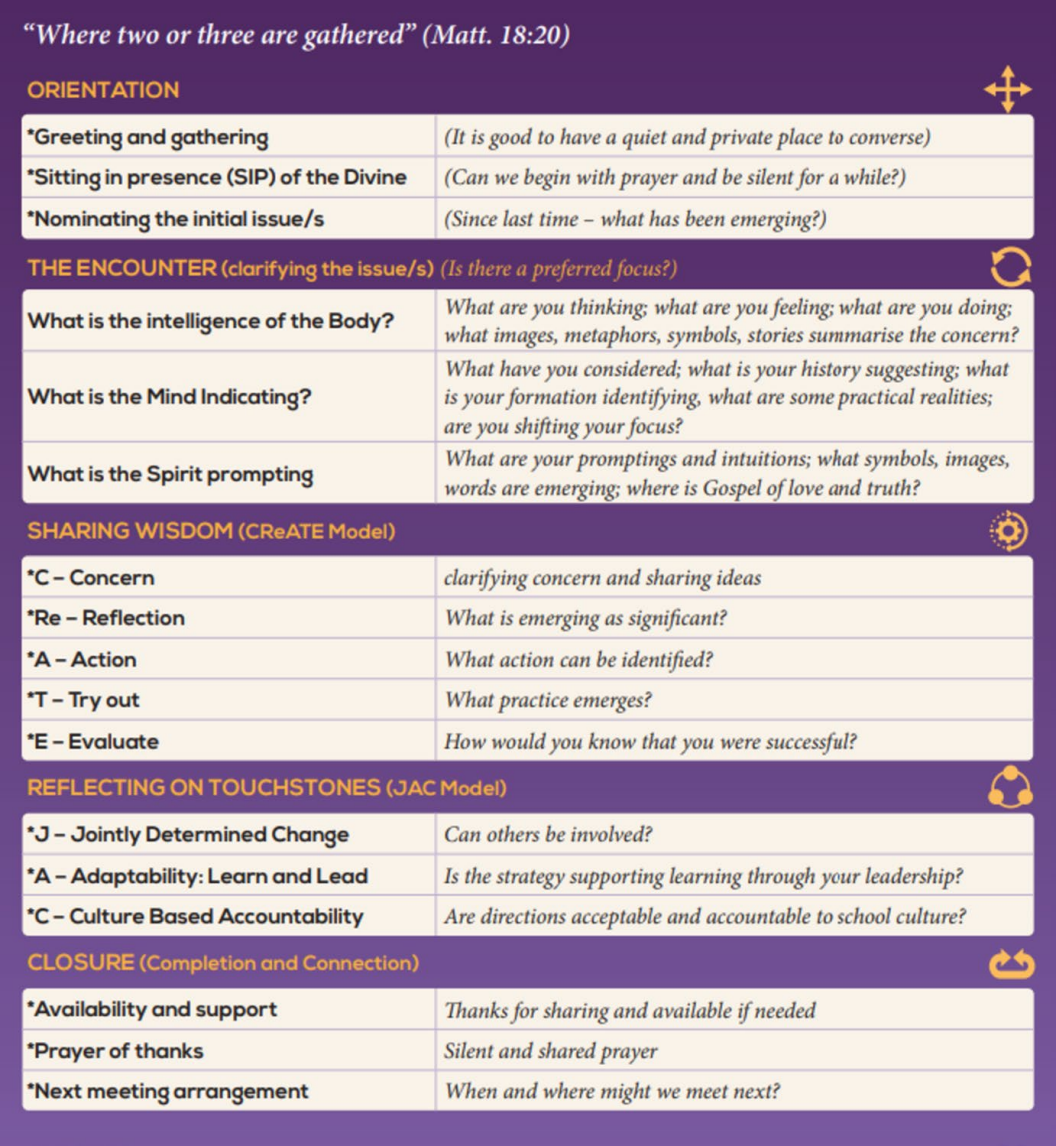
The conversation process

The characteristics of meaningfulness, manageability, and comprehensibility of the Mamre program presume capabilities of both the principal and companion in context awareness, understanding organizational parameters, and the arrangement of opportunities for “walking together” in a relationship inspired by the Spirit. This form of accompaniment is a distinctive process. It is defined within Mamre as a mutually empowering process informed by the Spirit and undertaken in a respectful, dialogical relationship between people of faith in support of mission. However, it may interact with and complement experiences of training (directed learning), coaching (modeling leadership skills), counseling (facilitating self-reflection), supervising (managing for accountability), consulting (advising on options), and spiritual direction (supporting the awakening of the Spirit). The relationship between the companion and the principal is primarily a process of journeying as an experience of encounter, centered in the incarnational presence of the Spirit and the search for wisdom in bringing a Christ consciousness to the ministry of leadership and the outcome of well-being in the Catholic school. As a mechanism for differentiating the uniqueness of Mamre, an advisory forum nominated principles, practices, outcomes, and overall perceptions as important to this accompaniment (see Fig. 1). It is from these understandings that the processes of formation and evaluation of the initiative were conceived. To support this process, a draft conversational schedule was prepared for companion use (see Fig. 2).

The research question which focused the evaluation of Mamre was: In what ways is the professional life and well-being of senior principals advanced through the accompaniment initiative? With sub-questions of:

1. What nominated factors influenced entry into the accompaniment initiative?
2. What design principles influenced the effectiveness of the accompaniment initiative?
3. What process factors contributed to the effectiveness of accompaniment?
4. What environmental circumstances influenced the effectiveness of accompaniment?
5. What nominated outcomes of the accompaniment initiative were valued by the participants?

## Method

### Program Development and Participants

The philosophy of Mamre was developed from collaborative discussions over six months among system authority leaders and consultant academics in a major Australian metropolitan city. It was informed by the relevant literature and was shaped in accord with the system authority’s priorities of supporting experienced principals in their professional well-being within established processes of support and decision-making practices. The vision for Mamre was to promote leadership sustainability, passion, and effectiveness in mission. The initiative operated outside of line management relationships and acted as a complementary support program to established organizational relationships. Central to the professional support was the wholistic well-being of the principal advocated by the social psychiatrist Antonovsky (1979) who argues health promotion as understanding and harnessing the factors that produce and maintain health, rather than what defines it. In this light, well-being is often viewed as an intrinsic part of health, and is multifaceted, comprising physical, mental, and social dimensions (Chopra & Tanzi, 2018).

The Mamre pilot study included six highly experienced principals with postgraduate qualifications and leadership experience in at least two Catholic schools. Principals are identified in the display of data as P1 to P6. Five of these principals were based in primary schools, with the six principals evenly distributed across schools with less than and greater than 500 students. The three companions, identified as C1 to C3, possessed substantial experience in leadership along with performance capabilities. Each companion worked with two principals. Additionally, two “others,” as representatives of the advisory forum, supported the formation, co-ordination, and supervision of companions. A formation program provided awareness, discernment, and professional learning for companions and principals.

### Data Collection and Analysis

Program evaluation involved a mixed methods pilot study using an online survey as part of the formation process, a mid-point progress report, and a final review using online survey. Quantitative data were collected using a five-point Likert scale with 1 indicating a very low level of agreement with the statement and 5 indicating a very high level of agreement. Qualitative comments drew responses on program appeal, interest, motivation, and challenge. A summary of the stages and processes of data collection and analysis is displayed in Table 1.

**Table 1:** Summary of the evaluation method

Stages	Participants	Data collection	Data analysis
Initial	6 × Principals 3 × Companions 3 × Others	Readiness survey assessment (quantitative and qualitative)	Initial perceptions thematic
Mid-point	3 Companions	Progress questions	Mid-point perceptions
Final	6 × Principals 3 × Companions 3 × Others	End of pilot review survey (quantitative and qualitative)	Final perceptions thematic

Data analysis employed descriptive statistics (Pallant, 2016) to analyze quantitative responses, supported by qualitative comments organized thematically (Smith et al., 1999). Overall, data analysis involved: Initial participant perceptions as to Appeal, Understanding, Motivation, and Challenge of the program; Mid-point observations reported by companions and gathered by the system authority; and program participant post program perceptions as to accompaniment principles, practices, and outcomes.

## Results

### Pre- and Post-Program Engagement

All participants responded positively to program appeal, understanding, motivation to engage, and the potential challenge of the pilot. Noteworthy were the high mean score changes as to program understanding and challenge from pre- to post-evaluations (see Table 2). No inferential statistical analyses were applied to the dataset as the participant numbers were small. Also important were the qualitative responses from participants as to the overall experience across the dimensions of appeal, understanding, motivation, and challenge.

**Table 2:** Pre- and post-program perceptions

Criteria	Stage	Survey items	Mean
Appeal	Pre	My level of appreciation with the scriptural imagination (Mamre) program is …	4.0
	Post		4.2
Understanding	Pre	My level of understanding of the overall pilot program is …	3.4
	Post		4.3
Motivation	Pre	My level of motivation for participation in the pilot program is …	4.5
	Post		4.8
Challenge	Pre	My level of challenge expected from the pilot program is …	3.8
	Post		4.2

#### Appeal

The initial high appeal of the program (mean 4.0), particularly with respect to the scriptural imagination of Mamre, was recorded in positive comments.

The idea of a safe space to discern and engage with the shared wisdom of the companion is appealing. Relationships are central to my role, and I feel that the quality of my relationships will be enhanced. (P3)

The appeal of the program was registered consistently with commentary in its final stages of implementation echoing earlier sentiments, this time by one of the companions.

It is good to journey with people through the faith aspect of our work. It is something we all share. Although, in my work as a companion, it is more “understood” than observed in lengthy discussions about personal spiritual matters. (C3)

#### Understanding

Understanding of the program grew with participation across the year (pre mean 3.4; post mean 4.3). Notwithstanding, the initial understanding of the program did evidence substantial awareness which encouraged participation. Initial comments by an experienced principal included:

Companioning in dialog with each other. Shifting the focus from managerial to personal and how to best develop this in my leadership. (P6)

End of program comments indicated appreciation and confirmation of expectations being met. These included:

The freedom to share without concerns about judgment or impact on future career steps. (P2)

And

Time with a companion, reflecting on my role as leader and the integration of spirituality and leadership. (P1)

#### Motivation

The initial motivation for participation (mean 4.5) underlined a commitment to mission and the opportunity for ongoing development and sustaining well-being. Within a context of high expectation, the mean score of 4.8 at the conclusion of the program reinforced its importance.

I saw it as an opportunity to reinvigorate my personal faith journey and strengthen my capacity to lead. I am hoping for an experience that reimagines the purpose of my role. Keep a fire burning. (P2)

Motivation continued to be positive and appreciative across the program as end point comments by an advisory forum member (other) included:

This is a unique program that is built on, recognizes the experience and potential of both, the companion and the principal. (O3)

And a principal commented:

Conversations that provide direction. (P4)

#### Challenge

Principals and companions perceived the initial challenge from the program as being relatively high (mean 3.8) with one comment representative of the group overall:

I think the challenge for me is how to use the learnings and experiences to engage in parish life and lead a complex, diverse school community. (P2)

The challenges perceived at the end of the program (mean 4.2) registered similar and more informed responses as one member of the advisory forum reported:

The power of connection and interaction will create new exploration points and areas of focus that respond to the participants’ reality. The program by its nature should aim to be dynamic and responsive. (O3)

#### Mid-point Observations

Companion perceptions were invited as to meeting frequency, location, engagement, and process. This qualitative feedback provided a focus on Mamre’s practical application.

*Frequency:* Both the principals and I have been very aware of the time limit of one hour, which works well. Anything longer is just general chitchat. (C1).

*Location*: Companion meetings have been held at both the school venues and in more casual coffee shops as a softer way to get to know the people involved. I have left the choice of venue up to the principals to suit their diary opportunities. (C1).

*Engagement*: The focus has been on leadership and their place in the school and the different personalities they transact with. (C2).

*Process*: The outline of the meeting, which was provided to me during the in-service, has proven to be incredibly supportive in giving shape to the meetings. The training days are essential. (C1).

## Overall Program Perceptions

At the conclusion of the program, both principals and companions rated the importance of the accompaniment principles, identified the most effective practices, and highlighted the enhanced outcomes.

### Accompaniment Principles

Principals and companions held positive perceptions that the principles of the program were relevant and had been reinforced in its delivery. Notably, for each of the program principles, the group of principals held more positive perceptions than the companions (see Table 3).

**Table 3:** Principal and companion perceptions of the accompaniment principles

Principles	Participant	% Response proportions					Mean
		**1**	**2**	**3**	**4**	**5**	
Graced and gifted	Principal				40	60	4.6
	Companion			25	50	25	4.0
Gospel imagination	Principal				60	40	4.4
	Companion			25	50	25	4.0
Missionary discipleship	Principal			20	40	40	4.2
	Companion				100		4.0
Respectful relationship	Principal					100	5.0
	Companion				25	75	4.8
Mutual transformation	Principal				40	60	4.6
	Companion			25	25	50	4.3

Commentary on the principles were consistently positive for principals.

Listening with a loving heart. Connection. Being objective (C4)

And

The accompaniment within a sacred relationship. It has proven to be the case. (P3)

### Accompaniment Practices

Principals and companions held positive perceptions for each of the program practices (Table 4), although there were some noticeable differences expressed between the two groups.

**Table 4:** Principal and companion perceptions of the accompaniment practices

Practices	Participant	% Response proportions					Mean
		**1**	**2**	**3**	**4**	**5**	
Sacred relationship	Principal				60	40	4.4
	Companion			50	25	25	3.8
Relationship skills	Principal			20		80	4.6
	Companion				75	25	4.3
Interiority	Principal			20	60	20	4.0
	Companion			50	50		3.5
Sharing wisdom	Principal				40	60	4.6
	Companion					100	5.0
Ethical practice	Principal			20	20	60	4.4
	Companion				75	25	4.3

Comments associated with the practice factors served to expand on the quantitative findings.

I feel that my vocation as a Christian leader is very much stronger for my participation. (P3)

And

"Missionary Discipleship" is a concept that gives great emphasis to being that is imaged on the good, gentle, emphatic, and revolutionary - always a good mix that ensures one trusts in Spirit arising. (C1)

### Accompaniment Outcomes

Overall, principals and companions held positive perceptions of the outcomes of the Mamre program, although the level of appreciation varied reflecting the context from which the participants made their observations (see Table 5).

**Table 5:** Principal and companion perceptions of the outcomes of Mamre

Outcomes	Participant	% Response proportions					Mean
		**1**	**2**	**3**	**4**	**5**	
Collaborative culture	Principal			20	40	40	4.2
	Companion				50	50	4.5
Mission of Catholic education	Principal			20	20	60	4.4
	Companion				50	50	4.5
Advancing learning	Principal			20	40	40	4.2
	Companion			25	50	25	3.8
Engagement professional	Principal			20	40	40	4.2
	Companion				100		4.0
Contemplative dialog	Principal			20	20	60	4.4
	Companion			50	50		4.5

The outcomes arising from the pilot program were positive. Not surprisingly, the criterion of advancing learning was registered more positively by the principals than the companions. Associated comments from principals included:

Being able to find resolutions to problems myself after discussion. (P5)

And

I loved this opportunity! The expertise, wisdom, and generosity of the companion made this an outstanding experience. I have looked forward to every meeting despite knowing I was being held to account for the actions I had committed to undertaking. The freedom to share openly and honestly within a safe, non-judgmental space has been life-giving and made a difference to my leadership. (P2)

## Discussion

Two imperatives influenced the development and delivery of Mamre: First was the recognition and response to the needs of principals: “the joys and the hopes, the griefs and the anxieties of the men [sic] of this age, especially those who are poor or in any way afflicted, and these are the joys and hopes, the griefs and anxieties of the followers of Christ” (Paul VI, 1965, para. 1). Second, “this split between the faith which many profess, and their daily lives deserve to be counted among the more serious errors of our age” (para. 43). In response, a wholistic synthesis is emphasized which connects the experience of context with the life of the individual “let there be no false opposition between professional and social activities on the one part and religious life on the other” (para. 43). The delivery of Mamre recognized school leadership as a challenging professional responsibility, a cause of stress for many incumbents that warranted additional support.

Advancing the relationship of body, mind, and spirit through accompaniment conversations in the interests of health and well-being is already the subject of considerable attention in mental health (Rosmarin & Koenig, 2020) and in neurotheology (Newberg, 2010). In addition, the challenge of trialing and implementing practices that address well-being salutogenetically reinforces the faith-life nexus in ways that offer new language and practices to advancing well-being (Francis, 2023), specifically in contexts of mission within faith-based schools. The effectiveness of the program provides a basis for accompaniment application at scale in that it offers meaningful contours (Grumme, 2023), derived from experiential circumstances within which new initiatives might operate and flourish. In this way, the program offers guard rails for similar initiatives and a compass to model possibilities (ter Avest, Sporre, and Pirner, 2023). Mamre’s contribution lies in supporting coherence in health and professional practice, being situationally appropriate, and engaging personal involvement (Meyer, 2023).

The relationship of body, mind, and spirit is one of the Spirit-influencing and instigating body and mind connections. This is an understanding and commitment to the concepts of integration and embodiment. It accords with sustaining well-being in encompassing life dimensions of Having: physical needs met; Doing: establishing personal meaning; Living: experiencing belonging and place; and Being: growth toward actualization (Kusisto, 2023). This is meaningful integration whereby “human consciousness, interiority, and subjectivity are only available as embodied; each of these expresses and communicates themselves through the body” (Lane, 2015, p. 66). Awareness and activation of the body, mind, and spirit is to encounter; to attribute meaning and nurture transformation. It is the call to wholeness, an invitation that is inclusive, non-coercive, non-judgmental, and creative. It is premised on being gifted, being thankful and aware that all life is part of the sacred whole, and ideally making choices from a higher place in ways that advance personal awareness and the common good. It is said to be characterized as the ultimate journey home and the acceptance of the conclusion that all is connected (Myss, 2021, 2017).

The challenge of well-being through self-awareness entails becoming attentive to one’s internal and external environment. It is the practice of “being” present to the Spirit in self and others, having some understanding of the body, mind, and spirit relationship and being less subordinate to circumstances, history, and sub-conscious programs that can limit wholeness, and being present in the present moment. This form of living has been described as “The Physics of Living” (Amundson, 2003), a response that invites being more connected to Spirit; being less constrained by time; being appreciative through language; being less engulfed by *apriori* planning, being collegial, being present, and respectful; being hope-filled and trusting; and being committed to action. It is an approach to living consciously that reflects the wisdom of Spirit and the understandings of science in a quantum world. However, becoming aware and examining one’s inner self is not necessarily obvious, and, nor is the processing of information in the conscious and unconscious mind balanced. Checking in with self and exploring the inner world of experience in both the now and its impact in the past is something that is normally experienced as unfamiliar. More common in a world where distractions and priorities are toward the external world, that of the interior dimension to living is potentially overlooked or low in the order of personal priorities. Addressing this challenge and the opportunities for wholistic engagement is the practice of accompaniment.

The exploration of accompaniment within a Christian perspective is the opportunity for personal and community growth, the means for happiness and completeness: “I have said these things to you so that my joy may be in you, and that your joy may be complete” (John 15:11). It is the development, integration, and application of a meaning system that is life-giving, life-wide, life-long, and life-enabling. It is an invitation to know more, experience more, give more, and so be more. It is the challenge to live consciously in ways that empower the body, engage the mind, and connect with spirit within self and others. This appreciation holds in relationship the traditions of the Church, aspirations within the community, and a personal commitment to continuing evolution that ultimately evolves into a new creation.

Foundational to Mamre was the identification of principles that underpinned the conversational process. Program developers and participants reinforced basic assumptions about the nature of the person as graced and gifted, the Gospel imagination that inspired mission and respectful relationships, the view of leadership as missionary discipleship, and the potential for mutual transformation from open, supportive, and dialogical conversations. Moreover, the comprehensibility of the experience was enlarged as principals and companions contributed their own unique “story” to the experience, while formation in mind–body connections reinforced the personal capacity for change. The reality of context was recognized through applying touchstones of importance which provided for the manageability of potential outcomes (see Fig. 2).

The experience of Mamre underscored the intricate nature of accompaniment and the vital role of formation, skill development, professional supervision, and evaluation in navigating its complexities. More specifically, the role of practicing conversational skills within a definable structure supported by language promptings was an important aspect of formation for the companions (see Fig. 2). In this regard, the initial inquiry of companions as to the process potentially going beyond “having coffee together” to a flexible yet sophisticated process of dialog was reinforced within the implementation phase of the program (see Fig. 2).

The experience of Mamre confirmed research findings on strategies that support principals through structured dialog (Spiro et al., 2007). These included the provision of high-quality training; data gathering about program efficacy; ensuring an adequate frequency and length of the initiative; resourcing personnel involved; and a focus on student learning while sustaining leadership. In addition, this study contributed additional insights. First, leadership accompaniment benefited from a collegial-based mission partnership comprising principals, companions, system authority, and a commissioned independent agency. Second, practical benefits involved the establishment of an advisory group and a coordinator for ongoing program delivery. Third, clearly defined responsibilities and processes for principals and companions were essential, particularly in clarifying the accompaniment relationships with respect to other support personnel and administrative practices. Fourth, targeted skill development for companions supported the quality of dialog with principals. Fifth, the application of touchstones for decision making offered a common and consistent platform aligned to system authority expectations. Finally, the accompaniment process engaged practices that prioritized the well-being of the principal and their exercising of professional agency.

## Limitations

The study was conducted in an educational metropolitan setting, and the sample size was small with a total of 12 participants including school principals, companions, and advisory forum members. While the size of this pilot study did reflect the invitational nature of the program, this sample size was mitigated by the three stages of data collection, the quality of responses, and the participation by highly experienced principals and companions. The second limitation was that the conversational process underpinning the accompaniment process, while effective, would have benefitted from additional skills practice. In this context, the initial assumption as to the level of base competencies of the companion and the adequacy of the introductory formation was challenged. Despite these limitations, there is merit in exploring further instances of accompaniment in support of principal professional well-being in the workplace.

## Conclusion

The invitation of Mamre is to engage mutually, respectfully, and fully in the work of the Spirit in the promotion of professional practice and well-being. While the process holds immediate benefit and relevance, it does not stand alone. The process underpins the far goal of the Catholic school to educate with Spirit, intention, and quality, and to do so empowered by distinctive leadership supportive of human flourishing. Additional research of Mamre would involve evaluation across a wider participant group, articulation of participant responsibilities, and development of the formation experience with emphasis on furthering an appreciation and practice of skills centered in dialog. The experience of Mamre, while an initiative within a specific educational authority within Australia, may well be applicable in addressing the leadership and well-being needs of principals within faith-based schools nationally and internationally. In addition, the application of the program for inexperienced and aspiring principals as well as middle managers within school settings is a strategy that supports both the practice of leadership and the promotion and sustaining of well-being in the workforce. One final consideration is the application of the program for wider health and welfare pastoral settings.
